# Reno-protective effects of GLP-1 receptor agonist and anti-platelets in experimentally induced diabetic kidney disease in male albino rats 

**DOI:** 10.22038/IJBMS.2022.65061.14494

**Published:** 2022-12

**Authors:** Amani M. El Amin Ali, Hamed M. Osman, Azaa M. Zaki, Olfat Shaker, Asma Mohammed Elsayed, Mostafa Yehia Abdelwahed, Rahab Ahmed Mohammed

**Affiliations:** 1 Physiology Department, Faculty of Medicine, Fayoum University, Fayoum, Egypt.; 2 Professor of Physiology, Physiology Department, Faculty of Medicine, Azhar University, Cairo, Egypt.; 3 Professor of Biochemistry, Biochemistry and Molecular Medicine Department, Faculty of Medicine, Cairo University, Cairo, Egypt.; 4 Histology Department, Faculty of Medicine, Fayoum University, Fayoum, Egypt.

**Keywords:** Cilostazol, Diabetic nephropathy, Dulaglutide, Metformin, Nitric oxide

## Abstract

**Objective(s):**

The prevalence of chronic kidney disease in diabetics is progressively increasing with an increased risk of fatal complications.

**Materials and Methods:**

Sixty male albino rats were used in the study, and type 2 diabetes mellitus were induced. Diabetic rats were divided randomly into 5 groups, the control diabetic group and 4 treated groups were treated with metformin (group3), dulaglutide (group 4), metformin & cilostazol (group 5), and the last group was treated with dulaglutide & cilostazol (group 6). At the end of the experiment, the weight of rats and systolic blood pressure were estimated. After overnight fasting, the serum levels of blood glucose, lipid profile, and kidney function were measured. After scarification, gene expression of eNOS and NFKB in kidney tissue were estimated and kidney tissues were examined for histopathology.

**Results:**

Diabetic rats showed a significant increase in body weight, blood pressure, serum blood glucose, lipid profile, and impaired kidney function. Metformin and dulaglutide are associated with a significant decrease in blood pressure, blood glucose level, serum lipid profile, and improved kidney function. These changes are associated with a significant increase in anti-oxidative markers, and decreased inflammatory and fibrotic markers, especially with the addition of cilostazol.

**Conclusion:**

Metformin and dulaglutide have been shown to ameliorate kidney damage in diabetics by stimulating the anti-oxidant defense system, normalizing kidney functional parameters, and improving histopathological changes. The addition of cilostazol to metformin or dulaglutide increased some of their anti-oxidants and anti-inflammatory properties.

## Introduction

Type 2 diabetes mellitus (T2DM) is one of the most prevalent medical conditions, affecting over 415 million people worldwide (1). It is caused by a combination of genetic, environmental, and lifestyle factors, all of which interact together to produce insulin resistance and β-cell dysfunction, leading to hyperglycemia (2). Diabetes mellitus is characterized by microvascular and macrovascular complications related to hyperglycemia severity and duration. Microvascular complications include diabetic nephropathy, diabetic retinopathy, and diabetic neuropathy (1). Diabetic kidney disease (DKD) is a worldwide danger because it causes end-stage renal disease (ESRD) which affects mortality in diabetic patients. Diabetic nephropathy (DN) is a disease characterized by diabetic glomerular lesions, pathological albuminuria, and loss of glomerular filtration rate (GFR) **(**3**).** DN patients have pathological structural and functional changes in the kidney such as mesangial expansion, thickening of the basement membrane, and nodular glomerulosclerosis in the glomeruli (4). Hyperglycemia is accompanied by significant changes in glucose and lipid metabolism as well as induction of oxidative stress (5), which is involved to a great extent in the development of DM complications as it promotes the activation of NF-κB, which leads to the activation of inflammatory cytokines such as TNF-α and IL-6, and cell apoptosis (5).

Metformin is the traditional first-line treatment to lower blood glucose levels in T2DM. It has anti-oncogenic, cardio-protective, and anti-inflammatory effects (6). Metformin can attenuate cyclosporine A-induced renal fibrosis in rats (7). Dulaglutide, a glucagon‐like peptide‐1 (GLP‐1) analog, is a once‐weekly GLP-1 receptor agonist (GLP‐1RA) used for the treatment of T2DM. It improves glucose metabolism by stimulating glucose-dependent insulin secretion and inhibiting the release of glucagon. It has beneficial effects on cardiovascular risk factors by improving obesity, hypertension, and lipid profile (8). Hyperglycemia affects gene transcription of coagulation factors; leads to a high level of plasma fibrinogen, coagulation factor VII, and coagulation factor XII; increased endothelial expression of tissue factor and factor VIIa complex activity, and a decrease in the anticoagulant protein tissue factor pathway inhibitor (TFPI) (9). Cilostazol (CTZ) is a specific PDE-3 inhibitor, acting through increase cAMP levels in tissues to prevent platelet aggregation, CTZ decreases superoxide production in tissues (10). In the present study, we prove the reno-protective effects of metformin and dulaglutide therapy through multiple mechanisms and whether addition of cilostazol to dulaglutide or metformin increases the reno-protective effects of both drugs in diabetic nephropathy.

## Materials and Methods

***Animals***

Sixty Adult male white albino rats with an average weight of 220–280 gm were used in the study. They were housed and maintained in an air-conditioned animal house, subjected to a 12:12 hr daylight/darkness and allowed free access to water. All the ethical protocols for animal treatment were followed and supervised by the animal facilities, Faculty of Medicine, Fayoum University. The experimental protocol was approved by the institutional animal care committee. 

***Experimental drugs***

*Streptozotocin drug*

Sigma-Aldrich Company (USA). It is presented in powder form, purity of more than 99 % to be dissolved in freshly prepared sodium citrate buffer pH 4.5.

*Metformin drug*

Cidophage, CID company for Pharmaceuticals and chemical industries), provided in Tablets each one containing (500 mg) of Metformin hydrochloride, a biguanide derivative. It is freely soluble in water. It was dissolved in distilled water before use.

*Dulaglutide*

(Trulicity, Eli Lilly and Company pharmaceuticals, USA) provided in injection pen, each pre-filled pen contains 1.5 mg of dulaglutide in 0.5 ml solution, glucagon-like peptide-1 receptor agonist (DPP - 4 inhibitors). It was injected once weekly intraabdominally in rats. 

*Cilostazol drug*

(Pletal, Otsuka Company, Japan) provided in Tablets each one containing 50 mg of cilostazol and phosphodiesterase - 3 inhibitor antiplatelet drug. It was dissolved in distilled water before use.

***Experimental design and groups***

Rats were divided into 2 groups the first group (10 rats) received standard rat chow while the second group (50 rats) received a high-fat diet (HFD; 20% protein, 60% fat, and 20% carbohydrates) for 14 days followed by intraperitoneal injection with a single low dose of streptozotocin (STZ, 45 mg/kg) to induce Type II DM (11). Both the low doses of STZ and HFD are essential elements to induce T2DM with insulin resistance. Two weeks following STZ injection, fasting blood glucose (FBG) levels were tested with a glucometer (Accu-Chek Meter; Roche Diagnostics GmbH, Germany) and the rats with high FBG (>11.1 mmol/l, 200mg/dl) were used for the subsequent experiments. Diabetic rats, included in the study, were randomly divided into** 5 **equal groups, with a total of Six groups, each group composed of 10 rats: Group 1: Control normal rats. Group 2: control diabetic rats: type II diabetic rats with no treatment given. Group 3: Diabetic rats treated with metformin (Cidophage, 70 mg/kg/day) (12) for 12 weeks. Group 4: Diabetic rats treated with Dulaglutide (Trulicity) (1.5 mg/kg BW /week) (13) for 12 weeks. Group 5: Diabetic rats treated with metformin + Cilostazol (pletaal) (5 mg/kg BW /day) (14) for 12 weeks. Group 6: Diabetic rats treated with Dulaglutide (1.5 mg/kg BW /week) + Cilostazol (pletaal) (5 mg/kg BW /day) (14) for 12 weeks. The long duration ensures diabetic complications.

***Measurement of body weight and arterial blood pressure***

At the end of the experiment, the body weight of all rats in all groups was recorded, and the systolic blood pressure of animals was measured by a noninvasive blood pressure monitor (ML 125 NIBP, AD Instruments, Australia) from the tail of conscious rats by the tail-cuff technique. The animals were strained in plastic tubes, warmed for 30 min at 28 °C in a thermostatically controlled heating cabinet (Ugo Basille, Italy) for better detection of tail artery pulse, the tail was passed through a miniaturized cuff and a tail-cuff sensor that was connected to an amplifier (ML 125 NIBP, AD Instruments, Australia). The amplified pulse was recorded during automatic inflation and deflation of the cuff. Systolic BP (cuff deflation pressure) was defined as the point at which the cuff pressure corresponds to the restoration of the first caudal artery pulse (15). 

***Sacrifaction and chemistry***

At the end of the experimental protocol, Blood samples were collected from retro-orbital sinus by heparinized capillary tubes under light ether anesthesia after overnight fasting for 12-14 hr. Blood was collected in centrifuge tubes and allowed to clot at room temperature and then centrifuged at 3000 rpm revolution per minute for 15 min, then sera were separated and stored at -20 °C up to the time of use. 24 hr of urine were collected from all rats. The rats were sacrificed. Kidneys were collected by making vertical midline abdominal incisions. Post removed adherent connective tissues, one kidney from every rat was stored by inserting it in liquid nitrogen and storing at -80 °C until gene expression by PCR, and the contralateral kidney was fixed in 10% formaldehyde for histopathological examination.

***Biochemical analysis of glucose, HbA1c, serum albumin, serum triglycerides, High-density lipoprotein (HDL), Low-density lipoprotein (LDL) urea, and creatinine***

They were assayed in serum samples by colorimetric determination method using commercially available kits obtained from Biodiagnostic, Egypt. Urinary albumin/creatinine ratio was estimated, (albumin mg/creatinine g in urine was measured) to help identification of kidney disease that can occur as a complication of diabetes. 

***Estimation of Insulin, NO, MDA, and GSH in the rat serum by ELISA techniques***

The fasting serum insulin and nitric oxide levels were detected in serum utilizing ELISA kits (DRG International Inc., Springfield, NJ, USA), and malondialdehyde and glutathione peroxidase levels were measured using an ELISA assay kit (Sunglong Bio tech, Hangzhou, China). 

***Estimation of transforming growth factor –B (TGF-β), collagen IV, fibronectin (FN), fork head box O1 (FOXO1), and PKP (Protein kinase P) in the kidney tissue by ELISA***

They were detected in kidney tissues utilizing ELISA kits (Calbiotech, Austin, USA). ELISA kits use the Sandwich-ELISA method. The Micro Elisa strip plate provided in this kit has been pre-coated with an antibody specific to *TGF-β, collagen IV, fibronectin* PKB, and FOXO1. All protocols followed the manufacturer’s instructions.

***Detection of endothelial nitric oxide synthase (e NOS) and nuclear factor kappa B (NFK B) gene expression in kidney tissues of rats by real-time PCR***

 RNA isolated kidney homogenate utilizing Qiagen tissue extraction kit (Qiagen, CA, USA). Isolated RNA, either purity or concentrations, were established by the NanoDrop 2000 Spectrophotometer (Thermo Scientific, USA) and kept at -80 °C. Reverse transcription of RNA was carried out utilizing QuantiTect reverse transcription kit (Qiagen) as described in the manufacturer’s protocol. Quantitative assessment of eNOS and NFKB gene expression by real-time PCR: the expression of eNOS and NFKB genes was estimated by RT-thermal cycler (MJ Research Inc, Watertown, USA) as explained in the manufacturer’s protocol. Glyceraldehyde-3 phosphate dehydrogenase (G3PDH) was employed as an internal control for data normalization. Primers utilized for PCR were eNOS gene Forward primer: 5′-CATGAGGCTCAGCCCCAGAAC-3′ and reverse primer: 5′-AGTCAATCCCTTTGGTGCTCAC-3′. NFKB gene Forward primer:5’-ACGAAAGCGCAAGAAATCCC -3’ Reverse primer: 5’-TAACTCAAGCTGCCTCGCC-3. 

***Histopathological examination of kidney tissues***

Kidney samples were collected into PBS and fixed overnight in 40 g/l paraformaldehyde in PBS (phosphate buffer saline) at 4 **°**C. Serial 5-µm sections of the cortex and the medulla of the kidney were stained with hematoxylin and eosin *(H&E)* and Periodic Acid Schiff *(PAS)* Staining. 

***Statistical analysis***

Data was tabulated, arithmetic means and standard deviation (SD) were calculated, after checking normality of distribution, analysis of variance (one-way ANOVA) and comparison between every two groups by *post Hoc* Tukey test, using the statistical SPSS software for Windows, Version 18 (SPSS Inc., Chicago, USA). Significance was detected at *P*<0.05.

## Results

***Results of body weight and arterial blood pressure in the studied groups***

 As shown in Table 1, control diabetic rats (group 2) showed a significant increase in body weight and systolic blood pressure compared with control normal rats (group 1) **(***P-value<*0.0001**).** Groups 3, 4, 5, and 6 treated with metformin, dulaglutide, metformin and cilostazol, dulaglutide and cilostazol respectively all showed a significant decrease in body weight and systolic blood pressure compared with control diabetic rats (group 2). Dulaglutide and cilostazol **(**group 6**)** showed a significant decrease in BW and blood pressure compared with other treated groups (groups 3,4,5) **(***P-value<*0.0001).

***Results of serum levels of blood glucose, HbA1C, and serum insulin in the studied groups***

As shown in Table 2, control diabetic rats (group 2) showed a significant increase in serum blood glucose, HbA1C, and serum insulin levels compared with control normal rats (group 1) (*P-value<*0.0001**).** Groups 3, 4, 5, and 6 treated with metformin, dulaglutide, metformin and cilostazol, dulaglutide and cilostazol respectively all showed a significant decrease in serum blood glucose, HbA1C, and serum insulin levels compared with diabetic control (group 2) **(***P-value<*0.0001**).** No significant change between treated groups (3, 4, 5, and 6 as regards HbA1C and serum insulin levels but with a significant decrease in blood glucose level in the metformin group **(**group 3**) **compared with groups 5 and 6 **(***P-value<*0.0001).

***Results of treatment on serum lipid profile (HDL, LDL, and TG) in the studied groups***

As shown in Table 3, control diabetic rats (group 2) showed a significant increase in serum triglycerides and LDL and significant decrease in serum HDL compared with control normal rats (group 1) **(***P-value<*0.0001). Groups 3, 4, 5, and 6 treated with metformin, dulaglutide, metformin and cilostazol, dulaglutide and cilostazol respectively all showed significant decrease in triglycerides and LDL, and significant increase in serum HDL levels compared with diabetic control (group2) **(***P-value<*0.0001). Group 4 treated with dulaglutide showed significant increase in serum HDL levels compared with metformin (group3) **(***P-value<*0.0001). Group 6 treated with dulaglutide and cilostazol showed significant decrease in serum triglycerides when compared with other groups (3,4,5)** (***P-value<*0.0001). 

***Results of kidney function tests in the studied groups.***

As shown in Table 4, control diabetic rats (group 2) showed significant increase in serum urea and creatinine levels with significant decrease in serum albumin compared with control normal rats (group 1) (*P-value<*0.0001). Groups 3, 4, 5, and 6 treated with metformin, dulaglutide, metformin and cilostazol, dulaglutide, and cilostazol respectively all showed significant decrease in serum urea and creatinine levels with significant increase in serum albumin levels compared with diabetic control (group2) (*P-value<*0.0001). There were no significant differences between treated groups (3, 4, 5, and 6) as regards urea and creatinine levels but there was a significant increase in serum albumin levels in group 6 when compared with group (3) (*P-value<*0.0001). The A/C ratio was calculated based on the albuminuria and urine creatinine levels. It was significantly increased in diabetic rats with a significant decrease in all treated groups. Group (6) showed a decrease in the A/C ratio in urine when compared with other treated groups **(***P-value<*0.0001).

***Results of oxidative markers*** ***Malondialdehyde (MDA) and anti-oxidant glutathione peroxidase (GSH) in all studied groups ***

As shown in Table 5, control diabetic rats (group 2) showed significant increase in the level of MDA with significant decrease in the level of GSH compared with control normal rats (group 1) (*P-value<*0.0001). Treated rats (groups 3, 4, 5, and 6) treated with metformin, dulaglutide, metformin and cilostazol, dulaglutide, and cilostazol, respectively all showed significant decrease in the level of (MDA) with significant increase in the level of glutathione (GSH) compared with control diabetic rats (*P-value<*0.0001). there were no significant differences between treated groups in the level of oxidative markers, MDA. Groups 5 and 6 treated with combined therapy showed a significant increase in the anti-oxidant GSH compared with the metformin group (group 3) (*P-value<*0.0001). Group 6 (dulaglutide and cilostazol) showed significant increase in GSH compared with group 4 treated by dulaglutide only (*P-value<*0.0001).

***Results of fibrotic markers (transforming growth factor –B (TGF-B), Collagen type IV, and fibronectin (FN)***

 As shown in Table 6, control diabetic rats (group 2) showed significant increase in the levels of TGF-B, collagen type IV, and FN compared with control normal rats (group 1) (*P-value<*0.0001). Treated rats (groups 3, 4, 5, and 6) showed a significant decrease in the levels of TGF-B, collagen type IV, and FN compared with control diabetic rats (group 2) (*P-value<*0.0001). Dulaglutide and cilostazol group (group 6) showed a significant decrease in the levels of TGF-B and collagen type IV compared with the metformin group (group 3) (*P-value<*0.0001). Group 6 (dulaglutide and cilostazol group) showed a significant decrease in the levels of TGF-B and collagen type IV compared with the dulaglutide group (4) (*P-value<*0.0001). 

***Results of FOXO (fork head box O1) and PKB (Protein kinase B) in all studied groups***

As shown in Table 7, control diabetic rats (group 2) showed significant increase in the level of FOXO1 and decrease in PKB compared with control normal rats (group 1) (*P-value<*0.0001). Treated rats (groups 3, 4, 5, and 6) showed a significant decrease in the levels of FOXO1 and an increase in PKB compared with control diabetic rats (*P-value<*0.0001). Group 6 showed significant decrease in FOXO1 compared with group 3. Group 6 treated with dulaglutide and cilostazol showed significant increase in PKB compared with group 3 treated with metformin (*P-value<*0.0001). 

***Results of levels of nitric oxide (NO), gene expression of endothelial nitric oxide synthase (e NOS), and nuclear factor kappa B (NFKB) in kidney tissue in all studied groups***

Control diabetic rats (group 2) showed a significant decrease in the serum level of NO and e NOS gene expression with a significant increase in NF-κB gene expression compared with control normal rats (group 1) (*P-value<*0.0001). Treated rats (groups 3, 4, 5, and 6) showed a significant increase in the serum level of NO and e NOS gene expression with a significant decrease in NF-κB gene expression compared with control diabetic rats (group 2) (*P-value<*0.0001). Group 4 treated with dulaglutide showed a significant increase in the serum level of NO with a significant decrease in NF-κB gene expression compared with the metformin group (*P-value<*0.0001). Group 6 treated with dulaglutide and cilostazol showed a significant increase in the serum level of NO and e NOS gene expression with a significant decrease in NF-κB gene expression compared with the metformin group (*P-value<*0.0001). Group 6 showed a significant increase in the serum level of NO compared with group 5 (Table 8).

***Hematoxyllin & Eosin (H&E)***

**Figure 1:**
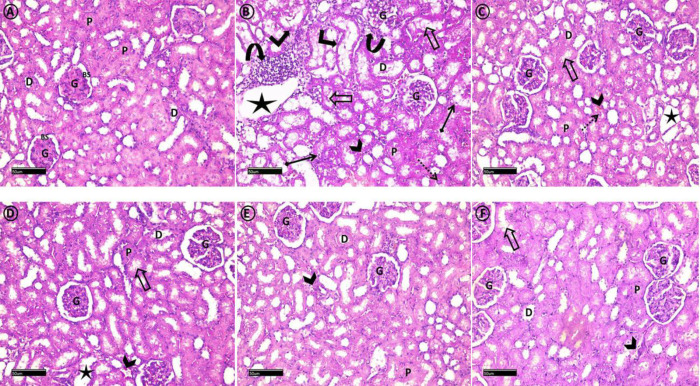
A photomicrograph of H&E stained sections of the renal cortex from all experimental groups: Control group (A), shows normal histological morphology of renal corpuscles with normal glomeruli (G) and Bowman`s spaces (BS). Proximal (P) and distal (D) tubules exhibit normal histological appearance. Diabetic group (B) shows fragmented glomeruli (G). Proximal (P) and distal (D) tubules appear distorted with epithelial cell vacuolization and detachment (diamond-ended arrows). Other lining cells have pyknotic nuclei (right-angled arrows). Cystic tubular dilation (star), intraluminal vacuolization (hollow arrows), and eosinophilic debris (dotted arrow) are obvious findings. Erythrocytes extravasation (arrowhead) and interstitial inflammatory infiltration (curved arrows) are distinct observations. Metformin (C) and Dulaglutide (D) treated groups show apparent normal renal cortex. Some glomeruli (G) exhibit fragmentation. Moderate tubular cystic dilation (stars), intraluminal vacuolization (hollow arrows), and eosinophilic debris (dotted arrow) are observed. Minimal erythrocytes extravasation (arrowhead) could be detected. Metformin + cilostazol (E) and Dulaglutide + cilostazol (F) treated groups exhibit apparent normal histology. Few glomeruli (G) show fragmentations. Residual intraluminal vacuolization (hollow arrows) and erythrocytes extravasation (arrowheads) could be noticed. (H&E stain, Scale bar=50 μm)

Examination of H&E stained sections revealed that control group (Figure 1A) exhibited normal histological architecture of renal corpuscles with normal glomeruli, Bowman`s spaces, Proximal and distal tubules. Diabetic group (Figure 1B) showed fragmented glomeruli. Distorted proximal and distal tubules were detected with their epithelial cell vacuolization and detachment. Other lining cells had pyknotic nuclei. Cystic tubular dilation, intraluminal vacuolization and eosinophilic debris were clear findings. Erythrocytes extravasation and interstitial inflammatory infiltration were distinct observation. Metformin (Figure 1C) and Dulaglutide (Figure 1D) treated groups revealed apparent normal renal cortex except for the existence of some fragmented glomeruli and moderate tubular cystic dilation, intraluminal vacuolization and eosinophilic debris . Minimal erythrocytes extravasation could be detected. Metformin + cilostazol (Figure 1E) and Dulaglutide + cilostazol (Figure 1F) treated groups exhibited apparent normal histology. Residual glomerular fragmentation, intraluminal vacuolization and erythrocytes extravasation could be noticed.

***Periodic acid schiff (PAS)***

**Figure 2:**
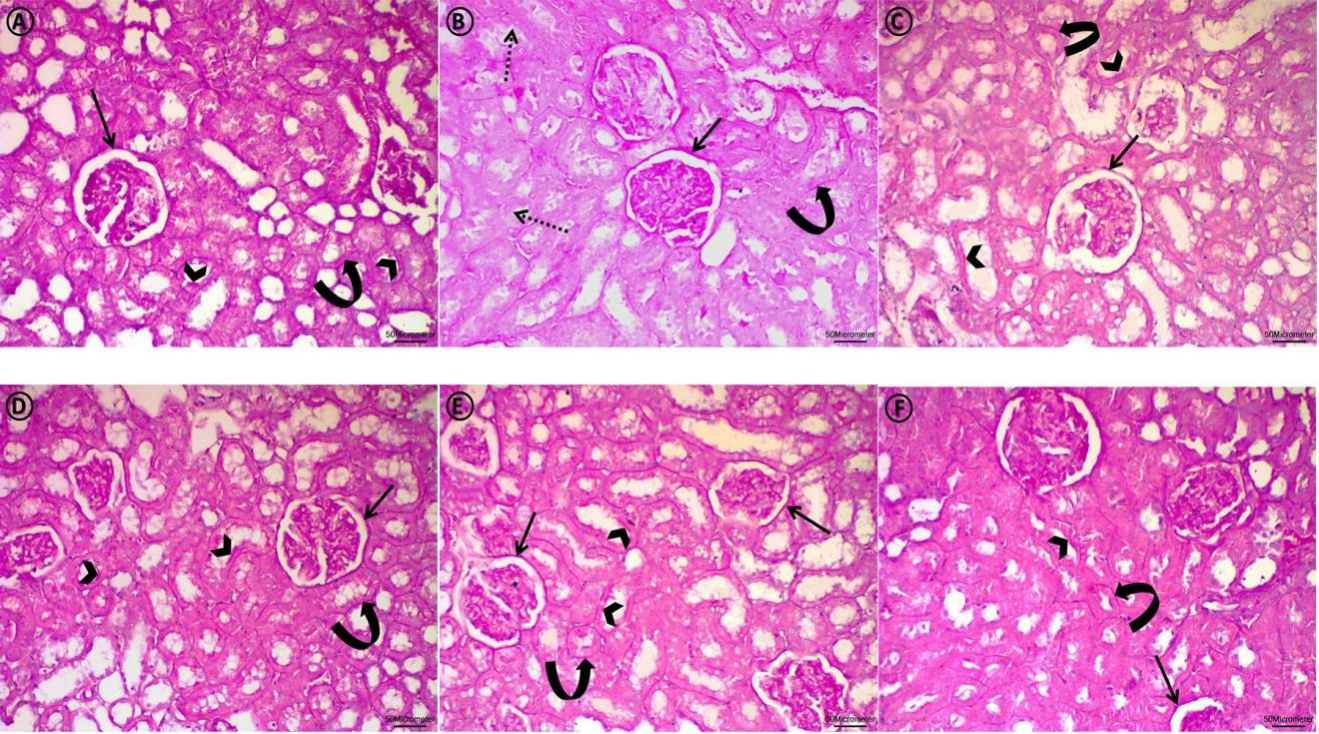
A photomicrograph of PAS stained sections of the renal cortex from all experimental groups: Control group (A), shows marked positive PAS reaction in Bowman capsular basement membrane (arrow). Brush border (arrowheads) and basement membrane (curved arrow) of most renal tubules exhibit strong reactions. Diabetic group (B) shows thickened Bowman capsular basement membrane (arrow) and disrupted tubular basement membrane (curved arrow) with obvious loss of tubular brush borders (dotted arrows). Metformin (C), Dulaglutide (D), Metformin + cilostazol (E), and Dulaglutide + cilostazol (F) treated groups exhibit strong PAS reaction in the basement membrane of Bowman`s capsule (arrows) and the majority of renal tubules (curved arrows). The intact tubular brush border (arrowheads) in most tubules is a clear finding. (PAS stain. Scale bar=50 μm)

Examination of PAS stained sections revealed that control group (Figure 2A) expressed marked positive PAS reaction in Bowman capsular basement membrane and both of brush border and basement membrane of most of renal tubules. Diabetic group (Figure 2B) appeared with thickened Bowman capsular basement membrane and disrupted tubular basement membrane with obvious loss of tubular brush borders. Metformin (Figure 2C), Dulaglutide (Figure 2D), Metformin + cilostazol (Figure 2E) and Dulaglutide + cilostazol (Figure 2F) treated groups revealed strong PAS reaction in basement membrane of Bowman`s capsule and the majority of renal tubules. Intact tubular brush border in most of tubules was a distinct finding.

## Discussion

Chronic kidney disease (CKD) is very commonly caused by type 2 diabetes. One of the key systemic microvascular difficulties of diabetes is Diabetic nephropathy (DN), which is extremely harmful to the health of patients (16). Glucagon-like peptide‐1 receptor agonists (GLP‐1 RAs) have good management of high blood glucose levels in different ways*. *Besides the glucose-lowering effect of GLP-1 RAs, many other mechanisms contribute to the preservation of renal functions (17). Phospho-diestrase inhibitor (PDE inhibitor) cilostazol increases the action of cAMP and cGMP signaling which are vital agents in cell development, survival, differentiation, and inflammation. Many studies use either metformin or GLP‐1 RAs alone. Here we investigate the effect of combined therapy of metformin with cilostazol compared with GLP‐1 RAs (dulaglutide) with cilostazol to show the effectiveness of combined therapy and the possible underlying mechanisms in renal protection of diabetic rats. 

In the present study, uncontrolled T2DM was associated with rising body weight, arterial blood pressure with very high serum glucose, and high insulin and Hba1C levels compared with control normal rats. The prolonged hyperglycemia in diabetic rats is associated with dyslipidemia in form of increased serum triglycerides and LDL with decreased HDL levels in the blood. These outcomes were granted by other studies done by Veneti *et al*. (18). In the present study, the treatment of diabetic rats with metformin represented a vital decrease in body weight, arterial blood pressure, serum glucose, insulin, and Hba1C levels as compared with the diabetic control group. The decrease in blood glucose level is associated with improvement of lipid profile in the form of decreased serum triglycerides and LDL with increased HDL levels in the blood. These findings are in agreement with *Derosa et al. *(19)*.* Metformin effect was explained as acting on multiple targets; reducing hepatic gluconeogenesis, intestinal glucose absorption, promoting pancreatic beta cell function, and improving insulin sensitivity. Metformin also decreases lipogenesis in the liver, muscles, and adipocytes and increases glucose utilization and GLP-1 secretion. *Figueiredo et al. *(20) reported that metformin affected hyperglycemia and hypertension (HTN) which are two main counter-regulators modulating DN in rats by inducing a significant increase in SOD, MDA, and urinary creatinine together with a significant decrease in serum insulin, glucose, triglycerides, total cholesterol, LDL, creatinine, urea, proteinuria, angiotensin II, and ABP. Moreover, metformin elevating plasma glucagon-like peptide-1 levels was another approach to normalizing plasma lipids.

The present study reported that treatment with dulaglutide in diabetic rats illustrated a key drop in body weight, blood pressure, glucose, serum insulin, and HbA1C levels as well as improved lipid profile in the form of decreased serum triglycerides and LDL with increased HDL levels in the blood in comparison with the diabetic control group. Secretion of insulin was stimulated by Glucagon‐like peptide‐1 (GLP‐1) receptor agonists in a glucose‐dependent manner. GLP‐1 receptor agonists inhibit the release of glucagon, improve glucose control and also weight loss, and had a lower risk of hypoglycemia **(**21**)**. GLP‐1 RAs led to weight loss mainly by decreasing body fat. GLP‐1 receptor agonists appeared to have an additive effect on the anorexigenic response of leptin, inducing satiety. *Greco et al. *(22) reported the presence of GLP-1R mRNA in the glomerulus and the initial part of proximal convoluted tubes, induced natriuresis and diuresis and inhibition of the sodium–hydrogen exchanger 3 (NHE3). This effect may have explained the lowering effect of blood pressure of GLP-1Rs agonists. GLP-1 has been indicated to raise B-cell mass through inhibition of apoptosis and encouragement of *β*-cell reproduction and neogenesis. *Tuttle et al. *(23) illustrated that once-weekly dulaglutide delayed the decrease in estimated glomerular filtration rate (eGFR), declined hyperglycemia, and dropped urine albumin/creatinine scale when compared with insulin. Our study also found that dulaglutide was superior to metformin in increasing serum HDL levels. The diabetic rats treated with dulaglutide and cilostazol showed a significant decrease in TGs compared with dulaglutide-treated rats, this combination had the best effect on serum TGS compared with each drug alone, most probably due to the combined effect of both drugs. Cilostazol inhibits cyclic nucleotide phosphodiesterase, potentiated its lipoprotein effects, and increased intracellular cAMP. The increased cAMP results in decreased plasma triglycerides (24). The present study demonstrated that the addition of cilostazol to dulaglutide does not affect the serum levels of glucose, insulin, and HbA1C, while the kidney function tests showed more improvement compared with dulaglutide alone. *Su et al. *(25) stated that pretreatment of cilostazol improved renal glomerular filtration function as confirmed by inhibition of the serum levels of creatinine and BUN, reduced the elevated level of MDA, and preserved that of GSH. Cilostazol might modulate vascular-related growth factors and oxidative stress molecules such as VEGF, PDGF, and nitric oxides (NO) (26). Data from the present study support that cilostazol treatment reduced serum lipids in HFD-fed rats besides their significant ability in suppressing weight. Cilostazol decreases lipid peroxidation and enhances levels of anti-oxidant enzymes in DKD rats. *Heeba et al. *(27) also recommended the use of combined cilostazol/metformin therapy over metformin monotherapy for a better effect on lipid profile status in high-fat-fed rats.

Our results showed that diabetic rats had huge raise in serum urea, creatinine, and a decrease in albumin levels in diabetic rats when compared with the normal control group. Metformin-treated diabetic rats showed a significant decrease in levels of urea and creatinine, and an increased level of albumin as compared with the diabetic control group. In accordance with our findings, *Mostafa et al.* (28) reported that diabetic mice with low dose metformin treatment showed decreased serum urea and creatinine levels, improved renal function, and ameliorated renal fibrosis observed with diabetic nephropathy. The results of the current work showed that diabetic rats treated with dulaglutide only showed a drop in urea and creatinine serum rates and increased albumin levels compared with the diabetic control group. These results are in agreement with Leon-Jimenez *et al. *(29) who reported that the renal benefits of GLP-1 RA were based mainly on glomerular filtration preservation and a reduction in macroalbuminuria. Dulaglutide is an effective nephron-protective drug for the treatment of patients with T2DM. The present study reported that dulaglutide was superior to metformin in increasing serum albumin levels and this was agreed by *Kristensen et al. *(30) who stated that treatment with GLP-1 RAs lowered the onset of macroalbuminuria development, fall in eGFR or increase in creatinine, ESRD, or renal mortality by 17%. The addition of cilostazol to metformin or dulaglutide decreases albuminuria better than each drug alone with better results than the combination of dulaglutide and cilostazol, this was agreed by *Su et al. *(25). The present results showed that diabetic rats had oxidative stress confirmed by decreased glutathione peroxidase** (**GSH), this was in agreement with *Zhang et al. *(31), who stated that induction of diabetes increased oxidative stress as marked by the high MDA and decreased GSH, CAT, and SOD levels. Our results also showed that metformin-treated rats showed an increased level of GSH compared with the diabetic control group, in accordance with our results, were *Huang et al. *(32) study on diabetic rats. Our results showed that dulaglutide treatment increased the level of GSH compared with the diabetic control group. The addition of cilostazol to dulaglutide causes a significant rise in GSH compared with dulaglutide alone. In agreement with the mentioned results***, ****Ragab et al. *(33) found that cilostazol ameliorated renal ischemia-reperfusion injury by restoring SOD and GSH levels and reducing MDA production. lCilostazol decreased lipid peroxidation and improved glutathione levels in hypercholesterolemic rats*.* Cilostazol normalized renal MDA, NO, GSH, and SOD which were disturbed by thioacetamide-induced nephrotoxicity in rats (34).

The results of the current study found that metformin treatment in diabetic rats showed a significant decrease in levels of fibrosis markers TGF- B, collagen type IV, and FN compared with diabetic control rats. These results are consistent with *Yi* *et al. *(35) who stated that metformin suppressed macrophage infiltration, inflammation markers expression, extracellular matrix proteins, TGF-β1 expression, and interstitial fibroblast activation, inhibiting TGF-β1 signaling pathways. Dulaglutide treatment in diabetic rats showed a significant decrease in levels of TGF-B, collagen type IV, and FN compared with diabetic control rats. These results are consistent with *Park et al.,* (36) who reported that (TGF)-β1, IL-1β mRNA, and protein expressions were significantly reduced by dulaglutide. GLP1 reduces inflammation in animals with diabetic kidneys via increased activation of cAMP–PKA signaling and inhibition of NADPH oxidase. GLP-1 receptor agonist attenuated oxidative stress, expression of TGF-β and fibronectin in the kidney, and decreased albuminuria via protein kinase A-mediated inhibition of renal NADP - oxidases. Cilostazol lowered collagen deposition and also lowered TGF-β which induced ECM accumulation that was shown to increase in HFD/STZ mice’s kidneys (37**).** The addition of cilostazol to dulaglutide in the current study caused a significant decrease in the levels of TGF- B and collagen type IV. This is in agreement with *Park et al.,* (37) who found that cilostazol functions to inhibit blood platelet accumulation, expand arteries, and resist multiple inflammatory factors. In addition, it had a preventive effect on diabetes chronic complications by preventing an enhancement of TGF-1 mRNA, which was a mechanism for mesangial accumulation of extracellular matrix in DN. 

Our study’s results found that diabetic rats showed a significant increase in FOXO1 serum levels, this goes with *Xu et al.* (38) who reported that over-expression of FOXO1 could cause mitochondrial dysfunction, including the production of ROS and cell apoptosis in diabetes. Metformin showed significant decrease in serum FOXO1 level compared with the diabetic control group. Metformin inhibits high glucose-induced mesangial cell proliferation, inflammation, and ECM expression through a Sirt1-FOXO1-autophagy axis (39). Diabetic rats treated with dulaglutide showed significant decrease in serum FOXO1 levels compared with the diabetic control group. GLP-1R decreased the FOXO1 mRNA expression and reduced renal phosphorylation levels of Akt and FOXO1 protein (40). The addition of cilostazol to metformin and dulaglutide caused a significant decrease in levels of FOXO1 compared with dulaglutide and metformin alone. This proves the anti-inflammatory functions of cilostazol.

Our results showed that induction of diabetes resulted in a decrease in the level of PKB (AKT) this goes with *Yuan et al.,* (41) who reported that ROS, associated with diabetes, directly increased inflammatory and adhesion factors expression, oxidized-low density lipoprotein formation, and insulin resistance. *Pan* *et al. *(42) reported that by increasing AMPK signaling and AMPK-independent pathways, metformin interferes with pathological diabetic nephropathy pathways to protect the kidneys from injury. Dulaglutide increased cAMP production and subsequently activated two downstream signaling levels of PKA and AKT signaling as shown by increased phosphorylated PKA (Thr197) and AKT (Ser473); As expected, the activated PKA signaling increased its target protein expression of HSF‐1, whereas the elevated AKT signaling suppressed the activation of NFκB (43). The addition of cilostazol to dulaglutide led to a significant increase in PKB (AKT) compared with dulaglutide only. Cilostazol induced suppression of NF-κB, inflammatory pathways, oxidative stress, and apoptosis through activation of multiple signaling pathways downstream of PI3K/Akt/eNOS, 

In our study, levels of NO and eNOS were significantly decreased in diabetic rats’ serum compared with the normal control group. This result was in agreement with *Jeddi et al. *(44) who reported significant decreases in mRNA expression of PI3K, Akt, and eNOS in diabetes. Metformin treatment in diabetic rats showed a significant increase in NO and eNOS serum levels compared with diabetic control rats. Metformin by its AMPK-dependent manner increased NO bioavailability. Our study found that dulaglutide treatment in diabetic rats showed a significant increase in NO and eNOS levels compared with the diabetic control group, these results are consistent with *Nauck et al. *(45). Endothelial cells express more eNOS, produced more NO, and suppressed endothelin formation. *Xiong et al. *(46) approved possible mechanisms underlying the vasorelaxant effect of GLP-1 in rats. Diabetes resulted in a significant elevation in nuclear factor κB (NFκB**) **compared with normal control rats, in agreement with our results were those of *El Din et al. *(47). Metformin suppressed apoptosis by inhibiting advanced glycation end product-mediated NF-κB activation and reactive oxidative species generation in renal tubular cells. * Nna et al. *(48) showed that mRNA levels of NF-κB were lower in the metformin group than in the control group. It was proved that by inhibition of advanced glycation end products (AGEs), oxidative stress, and NF-κb, metformin could suppress inflammatory responses, leading to a rise in NO bioavailability. Diabetic rats treated with dulaglutide showed a significant decrease in NF-κB compared with diabetic control rats, this was in line with* Li et al. *(49) who found that dulaglutide ameliorates the components of extracellular matrix (ECM), such as type II collagen and aggrecan, through down-regulation of matrix metalloproteinase MMP-13 and (MMP)-3, dulaglutide had a potent inhibitory effect against proinflammatory cytokine expression, as well as the production of reactive oxygen species (ROS). Finally, these effects of dulaglutide were mediated through the nuclear factor kappa-B (NF-κB) pathway. The combined therapy in the current study (dulaglutide and cilostazol) causes a significant increase in serum NO and eNOS expression with a significant decrease in NF-κB expression compared with dulaglutide only. Cilostazol possessed beneficial effects on diabetic nephropathy by means of regulating protein kinase C, TNF-α, TGF-β, and oxidative stress-relevant NF-κB activation **(**50**). **The histopathological examination of renal tissue of diabetic rats exhibited the presence of scattered atrophied hypocellular glomeruli with widened Bowman’s spaces, partial loss of brush borders and intra-tubular debris, marked interstitial inflammatory infiltrate, renal medulla showed collecting tubules with markedly edematous epithelial lining and intra-tubular hyaline casts, these findings were in agreement with* Furuichi et al. *(51). Diabetic rats treated with metformin or dulaglutide showed significant improvement in the kidney tissues compared with the diabetic control group seen as normal glomeruli with normal Bowman’s spaces, proximal tubules with mildly edematous epithelial lining and partial loss of brush borders, and renal medulla showed collecting tubules with mildly edematous epithelial lining. In line with our results were those of *Fang et al. *(52). Diabetic rats treated with combined cilostazol with either metformin or dulaglutide showed apparent normal histology, and few glomeruli show fragmentation. Residual intraluminal vacuolization with erythrocytes extravasation. The previous finding agreed with those of *Kim et al. *(53).

**Table 1:** Comparison between mean ± SD of body weight (BW) and systolic blood pressure in all studied groups of rats

**Groups**	**Normal control**	**Diabetic control**	**Metformin treated**	**Dulaglutide treated**	**Metformin + cilostazol treated**	**Dulaglutide + cilostazol treated**
**Data**						
Body weight **(gm)**	250±70	360±90+	260±75*	245±48*	255±59*#	230±51*@#$
Arterial blood pressure **(mmHg)**	92.2± 4.2	135.43±9.7+	105.1±5.3*	103.2± 2.2*	98.1±2.3*#	93.7±4.8*@#$

Data are expressed as mean ± standard deviation, and (*P*-value<0.05) is considered significant

+ Significant with the normal control group, * Significant with the diabetic control group, # Significant with the metformin-treated group, @ Significant with dulaglutide treated group, $ Significant with Metformin + cilostazol treated group

**Table 2:** Comparison between mean ± SD of serum level of glucose, insulin, and HbA1c in all studied groups of rats

**Groups** **Data**	**Normal control**	**Diabetic control**	**Metformin treated**	**Dulaglutide treated**	**Metformin + cilostazol treated**	**Dulaglutide + cilostazol treated**
**Serum glucose ** **(mg/dl)**	80.86± 8.51	386.22± 45.45 ^+^	117.39± 17.66 ^+*^	120.57± 24.35 ^+*^	142.27± 18.36 ^+*^#	130.60± 20.99 ^+*^#
**Serum insulin ** **(IU/ml) **	9.54 ±0.69	35.12± 3.56^+^	27.03±3.49^+*^	23.47±3.68^+*^	23.91±4.05^+*^	25±7.34^+*^
**Hb A1c** ** ( % )**	4.46±0.27	7.25±0.7^+^	4.9±0.44^*^	4.45±0.1^*^	4.8±0.8^*^	4.33±1.3^*^

Data are expressed as mean ± standard deviation, and (*P*-value<0.05) is considered significant

+ Significant with the normal control group, * Significant with the diabetic control group, # Significant with the metformin-treated group, @ Significant with dulaglutide treated group, $ Significant with metformin + cilostazol treated group

**Table 3:** Comparison between mean ± SD of serum levels of HDL, LDL, and TG in all studied groups of rats

** Groups** **Data**	**Normal control**	**Diabetic control**	**Metformin treated**	**Dulaglutide treated**	**Metformin + cilostazol treated**	**Dulaglutide + cilostazol treated**
**HDL** ** (mg/dl)**	52.33±1.07	25.2±8.3^+^	42.6±2.8+^*^	50.7±8.3^* #^	50.8±1.23^*^	45.02±0.01^* ^
**LDL** **(mg/dl)**	54.17±2.08	89.91±1.61^+^	50.96±1.95^+*^	52.16±1.6^*^	53.6±1.2^*^	50.02±1.3^+*^
**TG** **(mg/dl)**	136.6±4.4	236.43±3.9^+^	139.45±3.3^*^	135.3±2^*#^	142.5±1.2^+*@^	130.1±1.3^@*#$^

Data are expressed as mean ± standard deviation, and (*P*-value<0.05) is considered significant

+ Significant with the normal control group, * Significant with the diabetic control group, # Significant with the metformin-treated group, @ Significant with dulaglutide treated group, $ Significant with Metformin + cilostazol treated group

HDL: High-density lipoprotein; LDL: Low-density lipoprotein: TG: Triglycerides

**Table 4:** Comparison between mean ± SD of serum level of urea, creatinine, serum albumin, and albumin/creatinine ratio in urine in all studied groups of rats

** Groups** **Data**	**Normal control**	**Diabetic control**	**Metformin treated**	**Dulaglutide treated**	**Metformin + cilostazol treated**	**Dulaglutide + cilostazol treated**
Blood urea (mg/dl)	51.06±14.08	70.33±11.8^+^	55±9.8^*^	57.43±6.48^*^	56.82±11.43^*^	56.75±5.97^*^
Serum creatinine (mg/dl)	0.71±0.17	1.6±0.63^+^	0.81±0.3^*^	0.73±0.11^*^	0.66±0.1^*^	0.71±0.19^*^
Serum albumin (g/dl)	4.69±0.19	3.02±0.16^+^	4.00±0.03^+*^	4.69±0.03^+*^	4.53±0.04^+*^	4.82±0.03^*#^
Albumin/ creatinine ratio in urine (mg/g)	15.26 ± 6.58	113.4 ± 30.8^+^	55.1 ± 23.7^+*^	35.63 ± 5.20^+*#^	29.6± 2.5^*@^	25.63 ± 3.70^*#@$^

Data are expressed as mean ± standard deviation, and (*P*-value<0.05) is considered significant

+ Significant with the normal control group,* Significant with the diabetic control group, # Significant with the metformin-treated group, @ Significant with dulaglutide treated group, $ Significant with metformin + cilostazol treated group

**Table 5:** Comparison between mean ± SD of serum level of MDA and GSH in all studied groups of rats

** Groups** **Data**	**Normal control**	**Diabetic control**	**Metformin treated**	**Dulaglutide** ** treated**	**Metformin + cilostazol treated**	**Dulaglutide + cilostazol treated**
**MDA** ** (**U/ml**)**	0.34±0.04	0.75±0.06^+^	0.47±0.04^*^	0.36±0.03^*^	0.45±0.03^*^	0.36±0.06^*^
**GSH** **(**U/ml**)**	13.44±1.67	7.58±0.51^+^	10.27±0.89^+*^	11.74±1.78^+*^	12.21±1.1^*#^	13.33± 0.82^*#^**@**

Data are expressed as mean ± standard deviation, and (*P*-value<0.05) is considered significant

+ Significant with the normal control group, * Significant with the diabetic control group, # Significant with the metformin-treated group, @ Significant with dulaglutide treated group, $ Significant with Metformin + cilostazol treated group

MDA: Malondialdehyde; GSH: Glutathione peroxidase

**Table 6:** Comparison between mean ± SD of the levels of TGF-B, Collagen IV, and FN in all studied groups of rats

** Groups** **Data**	**Normal control**	**Diabetic control**	**Metformin treated**	**Dulaglutide treated**	**Metformin + cilostazol treated**	**Dulaglutide + cilostazol treated**
**TGF-B** ** (**PG/ml**)**	91.76±12.17	209.28± 10.17^+^	152.69± 17.77^+*@^	123.24± 9.51^+*#^	141.1± 15.84^+*@^	112.97± 11.12^+*#^**@**$
**Collagen** ** (type IV)** **(**pg/ml**)**	81.62±4.51	166.42± 5.68^+^	146.76± 6.7^+*^	145.74± 4.47^+*^	143.86± 4.23^+*^	137.89± 9.56^+*#^**@**
**FN** **(**ng**)**	3.19±0.5	4.64± 1.25^+^	3.19± 0.5^*^	3.19±0.4^*^	3.22±0.4^*^	3.22±0.5^*^

TGF-B: transforming growth factor –B; FN: Fibronectin

**Table 7:** Comparison between mean ± SD of serum level of FOXO1 and PKB in all studied groups of rats

** Group** **Data**	**Normal control**	**Diabetic control**	**Metformin treated**	**Dulaglutide treated**	**Metformin + cilostazol treated**	**Dulaglutide + cilostazol treated**
**FOX01** ** (pg/ml)**	45±4	112±24^+^	56±5^*^	54±6^*^	48±5^*^	44±8^*#^
**PKB** **(pg/ml)**	1187±54	545± 23^+^	1150± 34^*^	1165± 98^*^	1157± 67^*^	1176± 56^*^^#^

Data are expressed as mean ± standard deviation, and (*P*-value<0.05) is considered significant

+ Significant with the normal control group, * Significant with the diabetic control group, # Significant with the metformin-treated group, @ Significant with dulaglutide treated group, $ Significant with Metformin + cilostazol treated group

PKB: Protein kinase B; FOXO1: Fork head box O1

**Table 8:** Comparison between mean ± SD of levels of NO, eNOS, and NFKB in all studied groups of rats

** Group** **Data**	**Normal control**	**Diabetic control**	**Metformin treated**	**Dulaglutide treated**	**Metformin + cilostazol treated**	**Dulaglutide + cilostazol treated**
**NO **(Umol/ml)	20.92±1.98	6.35±1.24^+^	14.64±1.8^+*^	18.54± 1.87^+*#^	16.44± 1.63^+*^	20.79± 2.79^*#^$
**eNOS **(RQ)	0.76±0.06	0.32±0.07^+^	0.5± 0.06^+*^	0.59± 0.05^+*^	0.55± 0.19^+*^	0.66± 0.11^+*#^
**NFKB **(RQ)	3.3±1.11	7.5±0.75^+^	4.83±0.6^+*^	3.6±0.2^*#^	4.4± 0.26^*^	3.27± 0.76^*#^

NO: Nitric oxide; eNOS: Endothelial nitric oxide synthase; NFKB: Nuclear factor kappa B

## Conclusion

Diabetes affected the kidney via many pathways; oxidative stress, inflammation, fibrosis, and apoptosis. Both metformin and dulaglutide altered these pathways, favoring protection for the kidney. They ameliorate oxidative-induced kidney damage by lowering levels of lipid peroxidation and stimulating the anti-oxidant defense system. Also, to normalize the kidney functional parameters and decrease histopathological changes. The anti-oxidant and anti-inflammatory properties of cilostazol rendered it appropriate to mitigate the development of diabetic renal disease. The addition of cilostazol to metformin or dulaglutide increased some of the anti-oxidants and anti-inflammatory properties. 

## Authors’ Contributions

RAM and AMZ Conceived and designed the study; AMZ and AMEAA Processed and collected data, and performed experiments; AMZ, OS, and AME Analyzed and interpreted the results; AMZ and OS Helped with draft manuscript preparation and visualization; AMEAA, HMO, and MYA Critically revised or edited the article; AMEAA, HMO, and RAM Approved the final version to be published; AMEAA, H MO, and R AM Provided supervision; MYA did the major revision needed by reviewers; AME Interpretated the histopathological results. All authors approved the final version to be published, take responsibility for the content of the submitted manuscript, and made significant contributions to conceptualization and design, or acquisition, analysis, or interpretation of the data. 

## Funding

This research received no external funding.

## Conflicts of Interest

There were no conflicts of interest.
